# Association between CLOCK 3111 T/C polymorphism with ghrelin, GLP-1, food timing, sleep and chronotype in overweight and obese Iranian adults

**DOI:** 10.1186/s12902-022-01063-x

**Published:** 2022-06-02

**Authors:** Sara Rahati, Mostafa Qorbani, Anoosh Naghavi, Milad Heidari Nia, Hamideh Pishva

**Affiliations:** 1 Department of Cellular - Molecular Nutrition, School of Nutrition Sciences and Dietetics, Tehran University of Medical Sciences, PO Box: 14155-6447, Tehran, Iran; 2 Non-Communicable Diseases Research Center, Alborz University of Medical Sciences, Karaj, Iran; 3 Department of Genetics, Cellular and Molecular Research Center, Resistant Tuberculosis Institute, Zahedan University of Medical Sciences, Zahedan, Iran; 4 Cellular and Molecular Research Center, Resistant Tuberculosis Institute, Zahedan University of Medical Sciences, Zahedan, Iran

**Keywords:** CLOCK gene, Appetite, Food timing, Sleep, Chronotype, Obesity

## Abstract

**Background:**

Circadian Locomotor Output Cycles Kaput (CLOCK), an essential element of the positive regulatory arm in the human biological clock, is involved in metabolic regulation. The aim was to investigate the behavioral (sleep duration, food timing, dietary intake, appetite and chronobiologic characteristics) and hormonal (plasma ghrelin and Glucagon-like peptide-1 concentrations) factors that could explain the previously reported association between the CLOCK 3111 T/C SNP and obesity.

**Methods:**

This cross-sectional study included 403 subjects, overweight and/or obesity, aged 20- 50 years from Iran. The CLOCK rs1801260 data were measured by the PCR–RFLP method. Dietary intake, food timing, sleep duration, appetite and Chrono-type were assessed using validated questionnaires. Ghrelin and GLP-1 were measured by ELIZA in plasma samples. Participants were also divided into three groups based on BMI. Logistic regression models and general linear regression models were used to assess the association between CLOCK genotype and study parameters. Univariate linear regression models were used to assess the interaction between CLOCK and VAS, Food timing, chronotype and sleep on food intakes.

**Results:**

After controlling for confounding factors, there was a significant difference between genotypes for physical activity (P = 0.001), waist circumference (*P˂*0.05), BMI (˂0.01), weight (P = 0.001), GLP-1 (P = 0.02), ghrelin (P = 0.04), appetite (*P˂*0.001), chronotype (*P˂*0.001), sleep (*P˂*0.001), food timing (*P˂*0.001), energy (*P˂*0.05), carbohydrate (*P˂*0.05) and fat intake (*P˂*0.001). Our findings also show that people with the minor allele C who ate lunch after 3 PM and breakfast after 9 AM are more prone to obesity (*P˂*0.05). furthermore, there was significant interactions between C allele carrier group and high appetite on fat intake (Pinteraction = 0.041), eat lunch after 3 PM on energy intake (Pinteraction = 0.039) and morning type on fat intake (Pinteraction = 0.021).

**Conclusion:**

Sleep reduction, changes in ghrelin and GLP-1 levels, changes in eating behaviors and evening preference that characterized CLOCK 3111C can all contribute to obesity. Furthermore, the data demonstrate a clear relationship between the timing of food intake and obesity. Our results support the hypothesis that the influence of the CLOCK gene may extend to a wide range of variables related to human behaviors.

**Supplementary Information:**

The online version contains supplementary material available at 10.1186/s12902-022-01063-x.

## Introduction

Obesity is a complex and multifactorial chronic disorder. It is well-known that body weight is strongly influenced by genetic, behavioral, and environmental factors [1]. Among the environmental factors associated with obesity, great importance has been attributed to changing dietary patterns and physical activity. However, other changes that current lifestyles make to behavior, including sleep patterns, may be related to behavior. Indeed, regulating appetite and food consumption is influenced by sleep duration and sleep restriction, which in turn may increase the risk of obesity [2]. Common genetic effects are observed between insomnia, drowsiness, and obesity, which indicate a common genetic contribution to these phenomena [3]. In most living organisms, biological processes are organized through a 24-h cycle of day and night, indicating optimal physiological and behavioral timing. These rhythmic daily physiological adaptations known as ¨circadian rhythms¨ (circa = around; dies = one day), repeated every 24 h, are essential for coordinating vital processes [2]. This clock works by expressing several clock genes that may activate or deactivate the clock to display an overall 24-h pattern. The transcription factor encoded by the CLOCK (Circadian Locomotor Output Cycles Kaput) gene plays a key role in the mammalian circadian system. This transcription factor affects natural energy metabolism affecting various metabolic pathways such as glucose and lipid metabolism in target organs including muscle, liver, and adipose tissue [4]. Thus, some metabolic disorders are often associated with mutation in clock-related genes, which are explained by the altered expression of these transcriptional regulators, which in turn are key metabolic factors regulating nutritional behavior at the hypothalamic level and energy metabolism in peripheral tissues [5, 6]. Various studies have shown that CLOCK gene polymorphisms are associated with obesity and related diseases such as metabolic syndrome [7, 8]. A single nucleotide polymorphism entitled 3111 T/C (rs1801260) (Thymine to Cytosine nucleotide substitution Position 3111 of DNA sequence) was detected in the 3'-flanking region in the human CLOCK gene [9]. A previous study showed that people who are heterozygous or homozygous for the 3111 C allele have been reported to be more likely the evening preference and obese than those with the TT genotype [10, 11, 12]. Food is one of the external synchronizers of our environmental clocks. The main role of the circadian clock is to coordinate living beings with environmental signs. Changes in meal time lead to the separation of environmental oscillators from the central pacemaker. Thus, abnormal feeding time can disrupt the circadian system and cause unhealthy consequences in humans [13].

Due to the high prevalence of obesity, metabolic changes and changes in sleep time in the Iranian population, so far no study has evaluated the relationship between CLOCK polymorphism (rs1801260) and sleep duration, eating time, appetite, chronotype as well as the incidence of obesity in the Iranian sample. In addition, since most studies have been conducted on European populations, such a study on the Asian population also seems useful. Thus, our study helps to confirm and replicate these findings, as well as investigate whether health differences can be partly justified by differences in the genetic association of CLOCK genes with variables related to body weight regulation.

## Materials and methods

### Participants

The sample size was computed according to the following: n = [(Z_1-_$\mathrm{\alpha }/2$)^2^ × p(1-p)/d^2^], which p = 42% [14], d = 0.05 and α = 0.05, with 95% confidence and 80% power, 403 subjects were required in this cross-sectional study. The study population was collected from all regions of Zahedan, using community-based sampling and cluster sampling. Subjects were chosen based on the following inclusion criteria: aged 20 to 50 years, individual with overweight or obesity (BMI > 25 kg/m^2^ and BMI ˂ 40 kg/m^2^); Exclusion criteria: regular use of medications except contraceptives, a history of hypertension, cardiovascular disease, diabetes mellitus, liver and kidney disorders, hyperthyroidism, hypothyroidism, alcohol consumption, smoking, pregnancy or lactation, menopause, chronic diseases that affect a person’s diet, body weight fluctuations over the past 1 year, following patterns and special diets and non-routine diets and participants that their energy intake was less than 800 kcal or more than 4200 kcal. All subjects were genotyped for the near CLOCK rs1801260 (3111 T > C), using a Polymerase Chain Reaction-Restriction Fragment Length Polymorphism (PCR–RFLP) of their CLOCK genotypes. The data were collected from June to October 2019. The study was approved by Ethics Committee of Tehran University of Medical Sciences (NO: IR.TUMS.VCR.REC.1398.260). All participants were informed of the study nature and gave written consents. The study was conducted at the Department of Cellular-Molecular Nutrition, TUMS.

### Genotyping

At the beginning of the study, 10 cc blood was collected from each patient. Blood samples were collected after 12 h of overnight fasting in tubes with the anticoagulant, EDTA. Genomic DNA was extracted from the whole blood using the GeneAll, Exgene™Cell SV kit (Gene All, Korea) according to the constructor's protocol. DNA fragment containing a thymine-to-cytosine (T / C) substitution in CLOCK gene was genotyped by PCR–RFLP. The PCR amplification of the genomic DNA fragment for CLOCK was performed by the forward primer 5′- GGG AAA GTT CCA GCA GTT-3′ and reverse primer 5′- ATC CAG GCA CCT AAA ACA-3′( Copenhagen, Denmark) [15]. The PCR was performed in a total 20 μl containing 0.6 μl of each primer, 0.8 μl of DNA, 10 μl of Taq DNA Polymerase 2 × MasterMix (Ampliqon, Germany). The amplification protocol considered of an initial denaturation step at 95 °C for 5 min, followed by 35 cycles of denaturation at 95 °C for 30 s, annealing at 53 °C for 30 s, and extension at 72 °C for 30 s, and final extension at 72 °C for 5 min. The 3111 T/C polymorphism was identified by restriction of the 167 bp PCR-fragment with Bsp1286I*;* the C-allele is cut by Bsp1286I [16]*.* The digested product was then subjected to electrophoresis on 2% agarose gel (Boehringer Manheim GmbH, Mannheim, Germany), stained with green viewer (Pars Tous, Iran) and visualized on a Gel Doc-system (U.V.P Company, Cambridge, UK). A 50 bp Ladder (Fermentas, Germany) was used to determine the Length of the digested products. The T allele appeared as a 167 bp fragment after electrophoresis, whereas the C allele was cleaved by the registration enzyme and appeared as 38 bp and 129 bp fragments.

### Ghrelin and GLP-1

Blood samples were collected from all subjects in the morning, after 10 to 12 h of fasting and were centrifuged at 4 °C, and the plasma was stored at -80 °C for subsequent analysis. Plasma GLP-1 (Glucagon-like peptide-1) and ghrelin samples were measured by ELIZA (Enzyme-linked immunosorbent assay) method (Linco Research, St. Charles, MO). All samples for GLP-1 and ghrelin were run in duplicate.

### General, anthropometric and physical activity assessments

We collected general information, such as age, educational level, marital status, and history of weight loss in recent years using standard questionnaires. Weight and height were measured using the Seca scale (GMbH, Hamburg, Germany) with light clothing and no shoes on. BMI was calculated as the weight in kilograms divided by the square of height in meters. Waist circumference was measured midway between the iliac crest and the lower costal margin along with hip circumference. All anthropometric measurements were taken in accordance with World Health Organization standards [17]. The International Physical Activity Questionnaire (IPAQ) short form was used to assess physical activity, and was categorized as follows: low < 600, moderate (600–3500), and high (> 3500) (MET-h/wk). The reliability and validity of the IPAQ has previously been evaluated in Iranian adolescents [18].

### Dietary intake assessment

Dietary intakes were assessed by expert dietitians using a validated, 7-day food record [14]. Each subject reported the types and quantities of dietary intakes at six food occasions (breakfast, lunch, dinner and three snacks) and over-night, which was converted to grams per day using household measures. Total energy and dietary nutrients were assessed by the Iranian Food Composition Table (FCT) and N4 software.

### Chronotype questionnaire

Subjects fill out the 19-item morning/evening questionnaire (MEQ; score range: 16–86) of Horne and Ostberg, at the follow-up period. According to this score, individuals are categorized as neutral (53–64 of score), morning (above 64 of score) or evening (under 53 of score) types. Morning-evening typology is a way to characterize subjects depending on individual differences in waking/sleeping patterns and the time of the day people report to perform best. Some people are “night owls” and like to stay up late at night and sleep late in the morning (evening types), whereas others are “early birds” and prefer to go to bed early in the morning (morning-types) [19]. The reliability and validity of the MEQ questionnaire were assessed in Iranian population [20].

### Timing of food intake

The timing of food intake was self-reported via a 7-day food record. Specifically, participants recorded the start time, end time, and duration of individual food intake episodes during 5 weekdays and 2 weekend days. Participants were instructed and trained on how to accurately complete the food records at the beginning of the study, the data collected were then reviewed with a technician.

### Sleep duration

The duration of normal sleep was estimated using a questionnaire [12, 21]. The total duration of weekly sleep was calculated as [(min weekdays × 5) + (min weekend days’ × 2)]/7.

### Assessments of appetite

Visual Analogue Scales (VAS) of 100 mm were filled to assess the self-reported appetite sensations (satiety, fullness, hunger, and prospective foods consumption). The overall score of appetite suppression was calculated based on four appetite parameters using the following formula (satiety + fullness + [100 – hunger] + [100 – prospective food consumption])/4, with 0 indicating higher appetite/less satiety and 100 indicating lower appetite/more satiety (primary assessment of the self-reported appetite sensations) [22]. Reliability and validity of the VAS questionnaire were assessed in Iranian population [23].

### Statistical analysis

We used SPSS (version 25; SPSS Inc., IL) for statistical analysis of all data. Initially, the Kolmogorov–Smirnov test was used to assess the normality of the distribution. Hardy–Weinberg equilibrium and comparison of categorical variables were assessed by χ^2^ test. Participants of the study were divided into three groups: overweight, obese, and the total (overweight and obese) based on BMI. Then analysis of covariance (ANCOVA) analysis was used to compare the variables between various genotypes of polymorphism CLOCK (TT, CT, CC), which was adjusted based on age, sex, energy intake, and physical activity. Tukey’s multiple comparison method was employed to compare the different genotypes in terms of their parameters.

Any significant relationship between variables was further investigated through regression analysis. The relationship between appetite factors, sleep duration and food intake with different CLOCK genotypes in the study groups based on BMI was determined using multivariate linear regression. Linear regression results were reported as (β) coefficients and confidence intervals (95% CI). Also, the relationship between meal time, chronotype with different CLOCK genotypes in the study groups based on BMI was explored using logistic regression. The results of logistic regression were presented as odds ratio (OR) and 95% CI.

Logistic regression was used to investigate the relationship between meal time based on genotype and chronotype with the chance of obesity. In regression analyses, in the first model, the raw (unadjusted) relationship of variables with genotypes was checked and in the second model, this relationship for age, sex, energy intake, marital status, smoking status, occupation, education and physical activity was adjusted using Enter method.

In this study, we used univariate linear regression models to evaluate the interaction of CLOCK 3111 T / C * VAS, CLOCK 3111 T / C * Food timing, 3111 T / C * chronotype and CLOCK 3111 T/C * sleep on food intakes, which were adjusted according to age and sex (ANCOVA), if necessary. P-value ˂ 0.05 was considered statistically significant.

## Results

### Study population characteristics

The frequency of minor allele of CLOCK was 37%. The distribution of the CLOCK rs1801260 genotypes (TT, TC, and CC) was in the Hardy–Weinberg equilibrium (P = 0.76). The means and standard deviation (SD) of age, weight, BMI, and WC of individuals were 36.5 ± 8.7 years, 85.8 ± 10.6 kg, 30.2 ± 3.1 kg/m2, and 99.01 ± 8.8 cm, respectively.

### Association between population characteristics, biochemical parameters, behavioral parameters, and rs1801260 genotypes

A total of 403 Iranian subjects were classified based on BMI, and divided into three groups: overweight, obese and total papulation (Table 1). ANCOVA was used to evaluate the differences in anthropometric measurements, biochemical parameters and behavioral parameters across genotypes. The results of the study revealed that the mean of BMI, WC and physical activity (PA) in obese group and the total population were significantly lower in TT genotype, compared with individuals in the CT and CC genotypes (*P* < 0.05), after adjustment for age, sex, energy intake, and physical activity for BMI and WC, and age, sex and energy intake for physical activity.

**Table 1:** Characteristics of the study population across rs1801260 genotypes

Variables	Overweight (*n* = 221)				Obese (*n* = 182)				Total (*n* = 403)			
	Genotype			*P*-value	Genotype			*P*-value	Genotype			*P*-value
	TT (*n* = 99)	CT (*n* = 101)	CC (*n* = 21)		TT (*n* = 59)	CT (*n* = 91)	CC (*n* = 32)		TT (*n* = 158)	CT (*n* = 192)	CC (*n* = 53)	
**Demographic variables**												
Age(year)	36.4 ± 9	35.7 ± 9	34.2 ± 9	0.653	36.3 ± 8	37.1 ± 7	38.8 ± 6	0.477	36.5 ± 8	36.4 ± 8	37.1 ± 8	0.83
Physical activity (Met-minute/w)	1804 ± 1049	1531 ± 1002	1272 ± 858	0.052	1770 ± 1277^ab^	1129 ± 978^a^	825 ± 555^b^	**0.001**	1792 ± 1134^ab^	1334 ± 1008^a^	996.7 ± 713^b^	**0.001**
**Anthropometric factors**												
Weight(kg)	79.5 ± 7	80.7 ± 7	81.1 ± 10	0.491	90.8 ± 10	92 ± 8	95.7 ± 10	0.072	83.7 ± 10^a^	86.3 ± 9	90.1 ± 12^a^	**0.001**
Height (cm)	164.7 ± 7	165 ± 7.5	164.1 ± 9	0.36	165.1 ± 8	164.1 ± 6	164.8 ± 7	0.69	165.1 ± 7	164.6 ± 7	164.2 ± 8	0.59
BMI(kg/m^2^)	27.9 ± 1.1	27.5 ± 1.4	28.2 ± 1	0.06	32.1 ± 3.1^a^	32.6 ± 1.9	34.1 ± 3.2^a^	**0.01**	29.6 ± 2^a^	30.3 ± 2	31.6 ± 4^a^	**0.001**
WC (cm)	94.6 ± 6.7	94.2 ± 7.5	96 ± 6.9	0.627	102.1 ± 8^a^	104.6 ± 7	106.9 ± 7^a^	**0.017**	97.3 ± 8^a^	99.2 ± 9	102.7 ± 8^a^	**0.001**
**Food intake**												
Energy (kcal/day)	1869 ± 259^ab^	1946 ± 272^a^	2012 ± 238^b^	**0.033**	2486 ± 407^ab^	2527 ± 285^a^	2695 ± 486^b^	**0.048**	2096 ± 437^ab^	2232.4 ± 416^a^	2434 ± 526^b^	**˂0.001**
Carbohydrate (kcal/day)	322 ± 31^a^	326 ± 36^b^	304 ± 28^ab^	**0.037**	330 ± 54^a^	311 ± 38^b^	333 ± 44^ab^	**0.006**	310.1 ± 80^a^	322.8 ± 66^b^	355 ± 92^ab^	**0.001**
Protein (kcal/day)	86 ± 12	83.2 ± 12	81.5 ± 10	0.201	87.3 ± 22	79.3 ± 19	79.1 ± 21	0.075	82.8 ± 25.2	82.2 ± 20.5	87.9 ± 25	0.18
Fat (kcal/day)	63.1 ± 12^ab^	78.6 ± 18^a^	72 ± 17^b^	**˂0.001**	67.8 ± 12^ab^	73.8 ± 15^a^	84 ± 17^b^	**˂0.001**	62.5 ± 18^ab^	77 ± 24.2^a^	84.4 ± 22^b^	**˂0.001**
**Qualitative variables**												
Males(%)	58.2(53)	54.5(60)	66.7(12)	0.602	47.2(25)	49.5(50)	44.8(13)	0.895	54.2(78)	51.9(110)	53.2(25)	0.899
Married(%)	78(71)	72.7(80)	66.7(12)	0.056	88.7(47)	77.2(78)	96.6(28)	0.063	81.9(118)	75(159)	85.1(40)	0.080
University graduate (%)	61.5(56)	73.6(81)	72.2(13)	0.173	45.3(24)	63.4(64)	62.1(18)	0.086	55.6(80)	68.4(145)	66(31)	0.064
Current smoker (%) Occupation (%)	2.2(2)	1.8(2)	0	0.962	3.8(2)	6.9(7)	3.4(1)	0.182	2.8(4)	4.2(9)	2.1(1)	0.676
Unemployed	41.8(38)	38.2(42)	38.9(7)	0.981	49.1(26)	47.5(48)	51.7(15)	0.699	44.4(64)	42.9(91)	46.8(22)	0.909
Government employee	33(30)	31.8(35)	27.8(5)		22.6(12)	24.8(25)	24.1(7)		29.2(11)	28.3(11)	25.5(12)	
Worker	6.6(6)	7.3(8)	11.1(2)		9.4(5)	3(3)	3.4(1)		7.6(11)	5.2(11)	6.4(3)	
Self-employment	18.7(17)	22.7(25)	22(4)		18.9(10)	24.8(25)	20.7(6)		18.8(27)	23.6(50)	23.1(10)	

Variables are presented as mean ± SD for continuous variables and percent (%) for categorical variables. *P*-value is found by ANCOVA and adjusted for age, sex, physical activity, and total energy intake (residual method), except for dietary energy intake, which was only adjusted for age, sex and physical activity and for PA, which was only adjusted for age, sex and total energy intake. a, b: Significant difference between genotype by ANCOVA with Tukey's post hoc tests. Significant items with a *P* value ˂ 0.05 are bolded

There was also a significant difference between energy intake and rs1801260 genotypes in all study groups, where energy intake was lower in people with TT genotype than in those with CC and CT genotypes (*P* < 0.05). In addition, the carbohydrate and fat intake were significantly lower in people with TT genotype than in those with CC and CT genotypes among the study groups (*P* < 0.05).As shown in Table 2, there was a significant difference between rs1801260 genotypes and VAS in all study groups, where those individuals with genotype TT had a significantly higher satiety feeling than those with genotypes CC and CT (*P* < 0.05).There was a significant difference between rs1801260 genotypes and chronotype in all subjects; those with TT genotype were early sleepers and had a higher MEQ score than those with CC and CT genotypes (*P* < 0.05). Meanwhile, subjects with TT genotype had a significantly longer sleep time during the day than those with CC genotype (*P* < 0.05) (Table 2). There was a statistical significant difference between study groups at breakfast, lunch, and dinner times, where the participants with TT genotype ate their meals earlier than those with CT and CC genotypes, while those with CC genotype ate their meals later (*P* < 0.05) (Table 2).

**Table 2:** Behavioral and biochemical parameters of the study population across rs1801260 genotypes

Variables		Overweight (*n* = 221)				Obese (*n* = 182)				Total (*n* = 403)			
		Genotypes			*P*-value	Genotypes			*P*-value	Genotypes			*P*-value
		TT (*n* = 99)	CT (*n* = 101)	CC (*n* = 21)		TT (*n* = 59)	CT (*n* = 91)	CC (*n* = 32)		TT (*n* = 158)	CT (*n* = 192)	CC (*n* = 53)	
**Behavioral factors**													
VAS. score		46 ± 18^ab^	38 ± 19^a^	28 ± 11^b^	**0.009**	39 ± 19^ab^	25 ± 18^a^	17 ± 11^b^	**0.007**	43 ± 19^ab^	32 ± 21^a^	21 ± 18^b^	**˂0.001**
ME. score		63 ± 7.6^ab^	52 ± 6.9^a^	47 ± 6.8^b^	**˂0.001**	62 ± 8^ab^	50 ± 6^a^	47 ± 6^b^	**˂0.001**	63 ± 7.7^ab^	51 ± 7.7^a^	47 ± 6.8^b^	**˂0.001**
Sleep (h/day)		8.3 ± 1^a^	8 ± 0.9	7.7 ± 1.4^a^	**0.01**	8.5 ± 1^a^	8.1 ± 0.7	8 ± 0.6^a^	**˂0.001**	8.4 ± 1^ab^	8.1 ± 0.8^a^	7.6 ± 0.9^b^	**˂0.001**
Food timing (h)	Breakfast	7.7 ± 0.9^ab^	8.4 ± 0.9^a^	9.4 ± 1.2^b^	**˂0.001**	7.8 ± 0.8^ab^	8.9 ± 1.1^a^	9.2 ± 0.9^b^	**˂0.001**	7.8 ± 0.9^ab^	8.6 ± 1^a^	9.3 ± 1^b^	**˂0.001**
	Lunch	13.7 ± 0.7^ab^	14.2 ± 0.8^a^	14.9 ± 0.9^b^	**˂0.001**	13.7 ± 0.8^ab^	14.7 ± 0.9^a^	15.1 ± 1^b^	**˂0.001**	13.7 ± 0.7^ab^	14.5 ± 0.9^a^	15.1 ± 0.9^b^	**˂0.001**
	Dinner	20.2 ± 0.8^a^	20.8 ± 0.8	21.6 ± 0.6^a^	**˂0.001**	20.2 ± 0.8^ab^	21.2 ± 0.9^a^	21.4 ± 0.9^b^	**˂0.001**	20.2 ± 0.8^ab^	21 ± 0.9^a^	21.5 ± 0.8^b^	**˂0.001**
**Hormonal factors**													
GLP-1 (pg/ml)		47.3 ± 19	60 ± 16	42.1 ± 22	0.28	61.1 ± 16^ab^	54.1 ± 16.3^b^	38.8 ± 19^b^	**0.02**	53.3 ± 19^a^	56.4 ± 16	40.4 ± 20^a^	**0.02**
Ghrelin (ng/ml)		0.84 ± 0.4	0.8 ± 0.1	0.78 ± 0.5	0.64	0.87 ± 0.2^a^	0.92 ± 0.3	1.1 ± 0.9^ab^	**0.048**	0.86 ± 0.3	0.87 ± 0.2	0.94 ± 0.7	0.98

*VAS* Visual analog scale, *ME* Morning-evening type, *GLP-1* Glucagon-like peptide-1, *S* Short, *M* Medium, *L* Long; Variables are presented as mean ± SD for continuous variables and percent (%) for categorical variables. *P*-value is found by ANCOVA and adjusted for age, sex, physical activity, and total energy intake. ^a, b^ Significant difference between genotype by ANCOVA with Tukey's post hoc tests. Significant items with a *P* value ˂ 0.05 are bolded

Ghrelin hormone level was significantly lower only in obese persons with TT genotype than in those with CC genotype (*P* < 0.05). In addition, the GLP-1 hormone level was significantly higher in obese people with TT genotype than in obese subjects with CC and CT genotypes. Meanwhile, the GLP-1 hormone level was higher in persons with TT genotype than in individuals with CC genotype across the total population (*P* < 0.05) (Table 2).

### Association between CLOCK gene (rs1801260) and food intake, behavioral and biochemical parameters

Using general linear model (GLM), the association between CLOCK polymorphism (rs1801260) and obesity- related factors was investigated. For this analysis, subjects were classified based on BMI and divided into three groups: overweight, obese and total papulation. Then the TT genotype was considered as the reference group. The relationship between various appetite factors and rs1801260 genotypes was investigated through the multivariate linear regression analysis. After adjusting the model according to age, gender, energy intake, marital status, smoking, occupation, education, and physical activity, the satiety feeling level was significantly low in all study groups with CT and CC genotypes compared with TT genotype (*P˂*0.05) (Supplement Table 1).

In addition, ghrelin hormone level was 0.6 ng/ml higher in obese individuals with CC genotype than in those with TT genotype (Supplement Table 1). The GLP-1 hormone level in the total population and subjects with obesity with CC genotype was lower than in those with genotype TT by 11.15 and 22.5 pg/ml, respectively (Supplement Table 1). There was a significant association between rs1801260 genotypes and sleep duration in obese people; People with CT and CC genotypes had shorter sleep time than those with TT genotype (β: -0.47, 95%CI: -0.75, -0.19 and β: -1.04, 95%CI: -1.42, -0.66, respectively). In addition, in the total population, the subjects with CT and CC genotypes had shorter sleep time than those with TT genotype by 0.34 and 0.79 units, respectively (*P˂*0.05) (Supplement Table 1). The results of linear regression after adjusting of risk factors as a covariate in the model showed that genotype CC had a significantly higher energy intake than genotype TT in all participants (β:344.5, 95%CI: 195.1, 494), obese people (β: 226.1, 95%CI: 46.8, 405.3), and subjects with overweight (β: 200.5, 95%CI: 72.8, 328.2) (Supplement Table 1). The multivariate regression analysis of macronutrients after adjustment revealed that carbohydrate consumption in overweight subjects with CC genotype and obese people with CC genotype was 32.9 and about 22 units higher than those with TT genotype respectively (*P˂*0.05). In addition, fat intake in all study groups with CC and CT genotypes was significantly higher than those with TT genotype (*P˂*0.05) (Supplement Table 1). However, no significant correlation was found between protein intake and genotypes in study groups (*P > *0.05) (Supplement Table 1).

The results of post-adjustment logistic regression analysis showed that the chances of eating breakfast after 9 a.m. in overweight people with CT and CC genotype were 4 and 20% lower than those with TT genotype, respectively (*P˂*0.05). Moreover, this figure was 7% and 53% higher in obese individuals with CT and CC genotypes than in those with TT genotype respectively (*P˂*0.05). In addition, in all participants, the probability of delayed in eating breakfast was 5% and 34% higher in genotypes CT and CC than in genotype TT respectively(*P˂*0.05) (Table 3).

**Table 3:** Association of CLOCK variant rs1801260 with food timing and evening type

Group	Genotype	Breakfast		Lunch		Dinner		Evening type	
		OR(CI)	P^*^	OR(CI)	P^*^	OR(CI)	P^*^	OR(CI)	P^*^
Overweight	CT/TT	1.04(1.01, 1.16)	**˂0.001**	1.02(0.00, 1.14)	0.07	1.02(0.00, 1.20)	0.06	7.51 (-4.68, 20.98)	0.20
	CC/TT	1.20(1.05, 2.75)	**0.015**	1.20(1.06, 2.61)	**0.005**	1.14(1.01, 2.54)	**0.001**	37.77 (5.7, 247.6)	**0.002**
Obese	CT/TT	1.07(1.02, 1.23)	**˂0.001**	1.03(0.01, 1.92)	0.08	1.16(0.05, 2.04)	0.81	13.93 (-8.78, 146.8)	0.16
	CC/TT	1.53(1.20, 3.91)	**0.018**	1.43(1.16, 3.81)	**0.03**	1.34(1.03, 2.72)	**0.001**	53.53 (7.53, 380.2)	**˂0.001**
Total	CT/TT	1.05(1.02, 1.13)	**˂0.001**	1.02 (0.01,1.46)	0.06	1.08(0.03, 1.34)	0.08	12.59 (-5.60, 28.32)	0.09
	CC/TT	1.34(1.15, 3.23)	**0.005**	1.27(1.13, 3.57)	**0.001**	1.45(1.01, 3.79)	**˂0.001**	36.23 (9.76, 134.42)	**˂0.001**

TT genotype has 0 risk allele. CT genotype has one and CC genotype have two risk allele. TT genotype is considered as a reference

Morning type is considered as a reference. Breakfast before 9 AM, Lunch before 3 PM and Dinner before 9 PM are considered as reference

Binary Logistic Regression: ^*^adjusted model to age, sex, energy intake, marital status, smoking status, education, occupation and physical activity, as covariate; The results of the association are listed for categorical variables as OR (confidence interval) (OR (CI)) and Significant items with a *P* value ˂ 0.05 are bolded

Regarding the lunch time, our analyses showed that the chance of eating lunch after 3 PM in overweight people with CC genotype was 20% higher than in those with TT genotype. In addition, the chance of eating lunch after 3 PM in obese subjects with CC genotype and in all participants with CC genotype was 43% and 27% higher than in persons with TT genotype respectively (*P˂*0.05) (Table 3).

Similarly, overweight people with CC genotype were more likely to eat dinner after 9 pm, obese people with CC genotype, and all participants with CC genotype were 14%, 34%, and 45%, respectively, higher than those who had TT genotype (*P˂*0.05) (Table 3).

Multivariable-adjusted ORs and 95% CIs for evening type by rs1801260 genotypes are reported in Table 3. After adjusting for age, sex, energy intake, marital status, smoking status, education, occupation and physical activity, the chance of evening type in overweight people with CC genotype and obese persons with CC genotype and in all subjects with CC genotype was 37.7, 53.5, and 36.2 units higher than in those with TT genotype respectively.

### Interaction rs1801260 genotypes with chronotype, sleep, appetite and food timing on food intake

We used univariate linear regression models to evaluate the interaction of CLOCK 3111 T / C * VAS, CLOCK 3111 T / C * Food timing, CLOCK 3111 T/C * chronotype and CLOCK 3111 T/C * sleep on food intakes, which were adjusted according to age and sex (ANCOVA), if necessary.

**Fig. 1:**
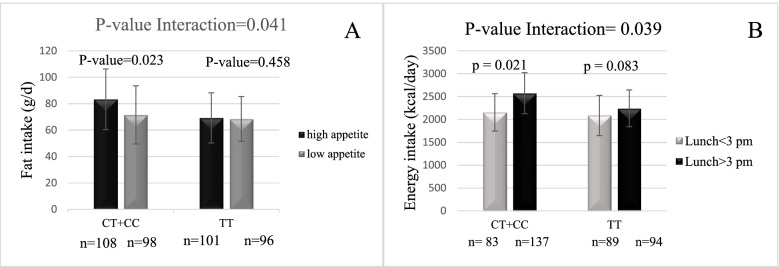
**A** CLOCK 3111 T/C SNP Interaction with appetite on fat intake. **B** CLOCK 3111 T/C SNP Interaction with lunch time on energy intake

The relationship between CLOCK 3111 T/C and appetite was also investigated in this study, whose results indicated a significant genetic interaction with fat intake. All participants were divided into two groups with high appetite and low appetite, and significantly different were found between genotypes at this locus (P _for interaction_ < 0.05). Among those with minor C allele, those with high appetite had a significantly higher fat intake compared with low appetite participants (β = 4.07, *P* < 0.05). However, no significant difference was observed between fat intake and high appetite as well as low appetite people TT genotype (β = 0.043, *P* < 0.05) (Fig. 1A).Regarding the interaction between CLOCK 3111 T/C genotype and lunch time, a significant relationship was found with energy intake (P _for interaction_ < 0.05); after dividing the participants into two groups of lunch eaters before 3 PM and after 3 PM, those with C allele who ate their lunch after 3 PM had a higher intake than those who ate their lunch before 3 PM (β = 353.8, *P* < 0.05). However, no significant relationship was found between lunch eaters before 3 PM and after 3 PM (β = 102.3, *P* < 0.05) (Fig. 1B). Interaction CLOCK 3111 T/C * chronotype and CLOCK 3111 T/C * sleep duration on food intakes was not significant (P _for interaction _> 0.05) (Supplemental Figs. 1 and  2).

### Association between late eating and risk of obesity based on genotype and chronotype

Logistic regression was used to investigate the relationship between food timing based on genotype, chronotype and the likelihood of obesity. The results showed that late lunch (after 3 PM) after adjusting covariates in morning type significantly increased the chance of obesity by 2.61 times (OR (95%CI) = 2.61 (1.20–4.71)) (Table 4). In addition, it was found that people with minor allele C who ate lunch after 3 PM and breakfast after 9 AM are more prone to obesity by 2.95 and 1.53 times, respectively (95% CI = 1.77–4.90, 1.32–1.89, respectively) (Table 4).

**Table 4:** Associations a between the timing of food intake and the odds ratios of obesity among people with a chronotype and genotype different

Food Timing	**Chronotype**	BMI					
		Crud			Model 1		
		OR	CI	*P*-value	OR	CI	*P*-value^*^
Breakfast (after 9 AM)	Morning	0.84	0.39, 1.80	0.65	1.34	0.27, 6.70	0.71
	Evening	0.86	0.42, 1.77	0.68	0.68	0.14, 3.13	0.62
Lunch (after 3 PM)	Morning	2.02	0.29, 13.6	0.47	2.61	1.20, 4.71	**0.003**
	Evening	0.72	0.21, 2.39	0.59	0.69	0.23, 1.99	0.42
Dinner (after 9 PM)	Morning	0.92	0.50, 1.67	0.79	0.49	0.14, 1.70	0.26
	Evening	0.87	0.28, 2.69	0.81	0.83	0.14, 4.87	0.84
	**Genotype**						
Breakfast (after 9 AM)	CT + CC	1.17	0.18, 7.64	0.86	1.53	1.32, 1.89	**0.01**
	TT	0.66	0.21, 2.07	0.47	0.72	0.31, 1.68	0.46
Lunch (after 3 PM)	CT + CC	1.57	0.25, 3.09	0.34	2.95	1.77, 4.90	**P˂0.001**
	TT	0.56	0.19, 1.62	0.28	2.19	0.63, 7.57	0.21
Dinner (after 9 PM)	CT + CC	1.53	0.84, 2.78	0.15	0.64	0.17, 2.33	0.50
	TT	1.72	0.85, 3.48	0.12	0.48	0.11, 2.13	0.33

TT genotype has 0 risk allele. CT genotype has one and CC genotype have two risk allele. Breakfast before 9 AM, Lunch before 3 PM and Dinner before 9 PM are considered as reference

^*^model adjusted for sex, age, energy intake, marital status, education, occupation, PA and smoking status. The results of the association are listed for categorical variables as OR (confidence interval) (OR (CI)) with the corresponding *P*-value

## Discussion

The aim of this study was to investigate the relationship between CLOCK gene rs1801260 polymorphism and energy intake, macronutrient intake, appetite-related factors, circadian rhythm, sleep duration as well as food timing in overweight and obese participants. One of the most important findings of our study is the effect of genotype on food intake time. Indeed, carriers of the minor C allele in all study groups consumed their food with a delay in all meals compared to carriers of the T allele. More interestingly, when we investigated the interaction between the eating time and the CLOCK rs1801260 on food intake, we found that eating lunch after 3 pm significantly increased energy intake in people carrying the C minor allele than in those carrying the T allele.

This is the first study to show that eating time with CLOCK 3111 T / C interacts with food intake. The results of a study by Ruiz-lozano et al. on eating time showed that people who are evening-type ate their lunch and dinner later than those who are morning-type [12]. Also, one study conducted by Garaulet in 2014 on obese people showed that less weight loss is observed in the group who had their lunch late (after 3 pm) than people who ate their lunch before 3 pm [13]. Studies conducted on experimental animals have shown that animals become obese when they eat at the "wrong time", though they seem to eat and consume the same amount of energy [24]. Regarding the results of previous studies and their emphasis on the role of chronotype plus eating time on obesity [25], in our study we challenged the effect of chronotype and genotype through eating time on obesity. According to our findings, both chronotype and genotype are associated with obesity, but genotype had a much stronger effect on the chances of obesity than chronotype. This is one of the latest findings on the effect of behavioral characteristics and genotype on obesity. The "time" of eating seems to be a factor in losing weight beyond "what" is eaten. Hence, the phrase "new treatment strategies should take into account not only the amount of calorie intake and the distribution of macronutrients, but also the time of food intake" must be realized and attention should be paid to the time of food intake based on genotypic, phonotypic characteristics and appetite rate of each person to observe the greatest impact of weight loss programs in obese and overweight people.

Furthermore, one of the interesting results of this study was the role of the body mass index (BMI) in the relation of research factors with CLOCK polymorphism. In overweight people, CC genotype had a relationship with food intake and behavioral factors except for sleep duration. In obese people, CC genotype had a relationship with food intake and behavioral factors, plus it had a significant relationship with sleep duration, Ghrelin and GLP-1 hormones, as well. This new finding should be considered in weight loss programs designed based on the genetic characteristics of individuals and BMI to see the most impact.

The results of our study support the notion that CLOCK locus (rs1801260) influences obesity-related behaviors, such as reduced sleep duration, evening-type, and appetite regulators such as plasma ghrelin. Successful obesity prevention is a major health care challenge. Behavior therapy has been suggested as part of weight loss programs. Its purpose is to facilitate the identification of stimulating factors for inappropriate behaviors that lead to weight gain, including excessive calorie intake. Behavior therapy is also used to create appropriate responses based on stimulating factors [21]. Identifying loci involved in the behavior may help achieve more effective and unique strategies [26]. Our findings suggest that the CLOCK 3111 T / C SNP can be effective in this regard. In particular, some studies have shown that carriers of the minor C allele experience less weight loss in the long-term than carriers of the T allele [21]. The study by Garaulet [21], matches the results of this study. It detonated that minor C allele is associated with evening type and reduction of sleep duration. Another study suggested that through evening type, the increase of Ghrelin plasma, and reduction of following the Mediterranean diet the combination of CLOCK and SIRT1 genotypes results in resistance to weight loss [27]. Ruiz-Lozano [12], revealed that people carrying CLOCK 3111 T/C polymorphism minor C allele who have undergone Bariatric surgery are mostly evening type. However, evening type have a higher weight and primary BMI than morning types. The morning types had a higher percentage of weight loss after surgery than the evening types. In addition, the evening types had reported a higher percentage of weight return four years after the surgery than the morning types. Mishima et al. [28] indicated a significant relationship between 3111C/C and staying up late at night. Various studies suggested that analyzing the circadian rhythm of people before the treatment is beneficial to predict their weight loss in the future [29, 30]. Maukonena [31] revealed that night owls are less likely to stick to the Baltic Sea diet (as a healthy diet). Mazri [32] demonstrated that night owls have habits such as eliminating breakfast, too much eating at night, lower protein and vegetable intake, as well as excessive Sucrose, sweet, coffee, and alcohol intake.

A number of mechanisms have been proposed to explain the association between reduced sleep time and obesity. For example, short period of sleep can lead to weight gain by increasing the time available for eating [33]. It has also been hypothesized that weight gain may be related to the reduced energy consumption due to increased fatigue and changes in temperature regulation, and most importantly the increase in appetite is due to changes in leptin or ghrelin levels [34]. In the present study, in line with studies of Ruiz-Lozano and Garaulet [12, 21], the level of ghrelin hormone was higher in obese people carrying C allele than T allele.

Epidemiological studies show a strong and significant relationship between short sleep and high ghrelin level. In this regard, ghrelin activates the neuropeptide Y (NPY) / Agouti-related protein (AgRP) mechanism and inhibits the proopiomelanocortin (POMC) / cocaine plus amphetamine-related transcripts (CART) neurons, and as a result, it creates a positive signal of pure nutrition [35]. At the same time, more support for ghrelin regulation by circadian clocks was obtained from rat models, where ghrelin would not be expressed rhythmically without other circadian clocks [36]. Therefore, it must be determined how the genes of the gastric clock regulate the synthesis and secretion of ghrelin. One of the new findings in the present study was GLP-1 hormone. GLP-1 hormone level was significantly higher in obese individuals carrying the major T allele than in those carrying the minor C allele. In a study conducted by Brubaker et Al. on GLP-1 as a missing link in the metabolic clock, GLP-1 was proposed as a new component of the peripheral metabolic clock. They demonstrated that the secretion of GLP-1 from intestinal L cells in humans and rats follows a rhythmic pattern which can be disrupted by factors such as constant exposure to light, eating the western diet, and eating at the wrong time. This can in turn disrupt the body's metabolic processes such as glucose intolerance and food intake [37]. In addition, based on Ranganath findings, high carbohydrate intake in individuals with the minor C allele may be another factor in lowering GLP-1 level [38]. The effects of two ghrelin and GLP-1 hormones on appetite are very interesting and significant; the VAS score in persons with minor C allele was lower than in persons with TT and the interaction of VAS and CLOCK 3111 T / C SNP on food intake showed that persons carrying C minor allele had a greater tendency to ingest fats than other food groups. Thus, the T allele seems to have a protective role against the feeling of hunger and overweight. Previous studies have shown that there is a relationship between chronotype of individuals and CLOCK SNP with carriers of C allele being more than evening-type carriers of T allele [27, 28, 39]. The results of our study are in line with previous studies.

A number of studies have suggested that eating habits in C allele carriers may be a key factor in preventing weight loss [40]. The results of our study showed that carriers of C minor allele had higher energy, carbohydrate and fat intake than carriers of T allele. There was no difference between protein intakes in study groups while the results of Garaulet study were completely different. They found that people carrying the C allele had higher protein intake than those carrying the T allele [21]. The reason for this difference may be different cultures and eating habits as well as the Spanish’s adherence to the Mediterranean diet compared to the Iranian population. Ruiz-lozano did not observe either any difference between food intakes among morning-type and evening-type people [12]. In our study, when we investigated the interaction of chronotype and genotype on food intakes, we did not observe any significant interaction between morning-type and evening-type participants.

Several limitations of the current study should also be considered. First, due to the cross-sectional nature of this study, causality cannot be inferred. Nevertheless, these results can provide hypothesis for prospective studies to evaluate and confirm the real causal relation. Second, since small sample size may limit statistical power, the results of the present study with a relatively small sample size should be interpreted with caution until replicated in large longitudinal studies. Third, underreporting of dietary intakes that are commonly observed in obese individuals, may lead to potential bias and null results [41]. However, subjects with extreme dietary intake values were not included in the analysis. On the other hand, current study as the same as other observational studies is prone to residual confounding due to unknown or unmeasured confounders [42]. Despite the limitations discussed above, this is the first attempt to study the association between CLOCK rs1801260 polymorphisms and behavioral and hormonal factors in in obese and overweight individuals and interaction between CLOCK rs1801260 polymorphisms and food timing, chronotype, sleep and appetite on food intake, according to our knowledge. Identifying these gene-behavioral factor interactions could be crucial in planning appropriate personalized nutritional advice for the prevention and management of obesity and its related consequences.

## Conclusion

As a result, CLOCK 3111 C carriers can experience changes in ghrelin and GLP-1 levels, followed by changes in appetite, reduced sleep duration, evening type, tendency to energy, carbohydrate and fat intake as well as delayed meal intake affect obesity. Our results support the hypothesis that the CLOCK gene may affect a wide range of variables related to human behaviors. Identifying clock genotypes in patients may help therapists identify the root causes of weight problems at individual level and contribute to a more personal and successful treatment. It is suggested that future trials are designed to achieve the best time to receive a meal based on genotypic, chronotypic, and behavioral characteristics for weight loss.

## Supplementary Information

**Additional file 1: Supplemental Table 1.** Association of CLOCK variant rs1801260 with behavioral and hormonal parameters. **Supplemental Fig. 1.** CLOCK 3111 T/C SNP interaction with chronotype on food intakes. **Supplemental Fig. 2.** CLOCK 3111 T/C SNP interaction with sleep on food intakes.

## Data Availability

The datasets used and/or analysed during the current study are available from the corresponding author on reasonable request.

## Abbreviations

AgRP: Agouti-related protein
ANCOVA: Analysis of covariance
BMI: Body mass index
CLOCK: Circadian Locomotor Output Cycles Kaput
CART: Cocaine plus amphetamine-related transcripts
CI: Confidence intervals
EDTA: Ethylene Diamine Tetra acetic Acid
ELIZA: Enzyme-linked immunosorbent assay
FCT: Food Composition Table
RFLP: Fragment Length Polymorphism
GLM: General linear model
GLP-1: Glucagon-like peptide-1
IPAQ: International Physical Activity Questionnaire
MEQ: Morning/evening questionnaire
NPY: Neuropeptide Y
OR: Odds ratio
PA: Physical activity
PCR: Polymerase Chain Reaction-Restriction
POMC: Proopiomelanocortin
SNP: Single nucleotide polymorphism
VAS: Visual Analogue Scales
WC: Waist circumference
