# Supplementary material for: Association between CLOCK 3111 T/C polymorphism with ghrelin, GLP-1, food timing, sleep and chronotype in overweight and obese Iranian adults
Source: BMC Endocr Disord. 2022 Jun 2;22:147. doi: 10.1186/s12902-022-01063-x (PMC9161580; doi:10.1186/s12902-022-01063-x)
Supplement: Supplementary file 1 — Additional file 1: Supplemental Table 1. Association of CLOCK variant rs1801260 with behavioral and hormonal parameters. Supplemental Fig. 1. CLOCK 3111 T/C SNP interaction with chronotype on food intakes. Supplemental Fig. 2. CLOCK 3111 T/C SNP interaction with sleep on food intakes. [file 12902_2022_1063_MOESM1_ESM.docx]

**Additional file 1:** **Supplemental Table 1**. Association of CLOCK variant rs1801260 with behavioral and hormonal parameters. **Supplemental Figure 1.** CLOCK 3111 T/C SNP interaction with chronotype on food intakes. **Supplemental Figure 2.** CLOCK 3111 T/C SNP interaction with sleep on food intakes.

Supplemental table 1. Association of CLOCK variant rs1801260 with behavioral and hormonal parameters.

| variables | Group | Genotype | Crude |  | Model 1 |  |
| --- | --- | --- | --- | --- | --- | --- |
|  |  |  | β (CI) | P | β (CI) | P^*^ |
| **Food intake** |  |  |  |  |  |  |
| Energy | Overweight | CT/TT | 53.02 (-18.04, 124) | 0.14 | 88.3(-36.8, 177.7) | 0.19^†^ |
|  |  | CC/TT | 101.03 (-28.2, 230.3) | 0.12 | 200.5(72.8, 328.2) | **0.002^†^** |
|  | Obese | CT/TT | -32.28 (-139.5, 74.9) | 0.55 | 72.61(59.1, 204.3) | 0.27^†^ |
|  |  | CC/TT | 182.26 (38.5, 325.9) | **0.01** | 226.1(46.8, 405.3) | **0.01^†^** |
|  | Total | CT/TT | 52.77 (-35.3, 140.8) | 0.24 | 95.2(-23.6, 171.4) | 0.07^†^ |
|  |  | CC/TT | 256.69 (121.7, 391.6) | **˂0.001** | 344.5(195.1, 494) | **˂0.001** |
| Carbohydrate | Overweight | CT/TT | 6.87 (-2.18, 15.93) | 0.13 | 2.34(-7.6, 12.2) | 0.64 |
|  |  | CC/TT | 19.96 (3.6, 36.33) | **0.01** | 21.9(3.8, 33.9) | **0.01** |
|  | Obese | CT/TT | 15.43 (-2.68, 33.56) | 0.09 | 0.58(-23.2, 22) | 0.95 |
|  |  | CC/TT | 21.68 (8.65, 34.71) | **0.001** | 22.02(5.5, 38.4) | **0.009** |
|  | Total | CT/TT | 6.03 (-13.80, 19.73) | 0.12 | 7.27(-1.2, 16.7) | 0.11 |
|  |  | CC/TT | 0.71 (-11.40, 12.83) | 0.90 | 3.79(-10.8, 17.2) | 0.52 |
| Protein | Overweight | CT/TT | -2.03 (-5.41, 1.34) | 0.23 | -2.01(-5.60, 1.58) | 0.27 |
|  |  | CC/TT | -2.96 (-9.12, 3.19) | 0.34 | -3.28(-9.80, 3.23) | 0.32 |
|  | Obese | CT/TT | -5.07 (-11.37, 1.07) | 0.10 | -4.35(-11.77, 3.07) | 0.24 |
|  |  | CC/TT | -2.93 (-11.37, 5.49) | 0.49 | -5.37(-15.61, 4.87) | 0.30 |
|  | Total | CT/TT | -3.65 (-6.98, -0.31) | **0.03** | -3.54(-7.26, 0.16) | 0.06 |
|  |  | CC/TT | -3.30 (-8.51, 1.90) | 0.21 | -4.24(-10.07, 1.57) | 0.15 |
| Fat | Overweight | CT/TT | 3.35 (-0.67, 7.39) | 0.10 | 5.55(1.33, 9.78) | **0.01** |
|  |  | CC/TT | 12.89 (5.71, 20.08) | **˂0.001** | 15.7(8.03, 23.3) | **˂0.001** |
|  | Obese | CT/TT | -0.39 (-7.57, 6.79) | 0.91 | 16.2(10.3, 22.2) | **0.03** |
|  |  | CC/TT | 12.07 (7.10, 17.04) | **˂0.001** | 8.94(0.71, 17.1) | **˂0.001** |
|  | Total | CT/TT | 7.44 (4.28, 10.60) | **˂0.001** | 9.98(6.47, 13.4) | **˂0.001** |
|  |  | CC/TT | 5.02 (0.002, 10.04) | **0.05** | 9.96(4.46, 15.4) | **˂0.001** |
| **Behavioral factors** |  |  |  |  |  |  |
| Appetite | Overweight | CT/TT | -5.28 (-10.63, 0.07) | **0.05** | -7.12 (-12.54, -1.69) | **0.01** |
|  |  | CC/TT | -13.06 (-22.73, -3.38) | **0.008** | -14.29(-24.13, -4.44) | **0.005** |
|  | Obese | CT/TT | -5.83 (-11.98, 0.31) | 0.06 | -10.07(-16.99, -3.16) | **0.005** |
|  |  | CC/TT | -13.32 (-21.55, -5.09) | **0.002** | -14.69(-24.24,-5.14) | **0.003** |
|  | Total | CT/TT | -6.26 (-10.44, -2.07) | **0.003** | -7.94(-12.15, -3.73) | **˂0.001** |
|  |  | CC/TT | -15.22 (-21.63, -8.81) | **˂0.001** | -13.77(-20.37, -7.17) | **˂0.001** |
| Sleep duration | Overweight | CT/TT | -0.22 (-0.5, 0.04) | 0.09 | -0.27(-0.55, 0.002) | 0.052 |
|  |  | CC/TT | -0.42 (-0.92, 0.07) | 0.09 | -0.49(-1.00, 0.008) | 0.054 |
|  | Obese | CT/TT | -0.09 (-0.35, 0.15) | 0.44 | -0.47(-0.75, -0.19) | **0.001** |
|  |  | CC/TT | -0.61 (-0.95, -0.27) | **˂0.001** | -1.04(-1.42, -0.66) | **˂0.001** |
|  | Total | CT/TT | -0.16 (-0.35, 0.02) | 0.08 | -0.34(-0.54, -0.15) | **˂0.001** |
|  |  | CC/TT | -0.52 (-0.81, -0.23) | **˂0.001** | -0.79(-1.10, -0.49) | **˂0.001** |

Supplemental Table 1 (continued)

| variables | Group | Genotype | Crude |  | Model 1 |  |
| --- | --- | --- | --- | --- | --- | --- |
|  |  |  | β (CI) | P | β (CI) | P^*^ |
| **Biochemical factors** |  |  |  |  |  |  |
| GLP-1 | Overweight | CT/TT | 15.17 (1.73, 28.61) | **0.02** | 9.52 (-7.53, 26.58) | 0.26 |
|  |  | CC/TT | -10.47 (-23.45, 2.5) | 0.11 | -0.20 (-18.1, 17.7) | 0.98 |
|  | Obese | CT/TT | 4.92 (-6.87, 16.72) | 0.40 | -8.64 (-22.6, 5.39) | 0.22 |
|  |  | CC/TT | -18.25 (-29.32, -7.18) | **0.002** | -22.6 (-38.2, -7.04) | **0.006** |
|  | Total | CT/TT | 9.59 (0.94, 18.23) | **0.03** | 1.27(-9.01, 11.55) | 0.80 |
|  |  | CC/TT | -14.4 (-22.7, -6.10) | **0.001** | -11.15(-21.5, -0.73) | **0.03** |
| Ghrelin | Overweight | CT/TT | -0.01 (-0.30, 0.28) | 0.94 | -0.03(-0.38, 0.31) | 0.82 |
|  |  | CC/TT | -0.04 (-0.32, 0.23) | 0.74 | -0.12(-0.49, 0.24) | 0.49 |
|  | Obese | CT/TT | -0.07 (-0.44, 0.29) | 0.67 | -0.03(-0.48, 0.42) | 0.88 |
|  |  | CC/TT | 0.52 (0.17, 0.75) | **0.004** | 0.60(0.40, 0.96) | **0.01** |
|  | Total | CT/TT | -0.02 (-0.26, 0.20) | 0.82 | -0.08(-0.35, 0.18) | 0.54 |
|  |  | CC/TT | 0.07 (-0.15, 0.31) | 0.50 | -0.11(-0.38, 0.16) | 0.42 |

TT genotype is considered as a reference. GLM; crude model and * adjusted model to sex, age, energy intake, marital status, education, occupation, PA and smoking status. †Model adjusted for sex, age, marital status, education, occupation, PA and smoking status. Significant items with a P value ˂ 0.05 are bolded.

[Grab your reader’s attention with a great quote from the document or use this space to emphasize a key point. To place this text box anywhere on the page, just drag it.]A

Supplemental Figure 1. CLOCK 3111 T/C SNP interaction with chronotype on food intakes

Supplemental Figure 2. CLOCK 3111 T/C SNP interaction with sleep on food intakes
